# Combined analysis of eIF4E and 4E-binding protein expression predicts breast cancer survival and estimates eIF4E activity

**DOI:** 10.1038/sj.bjc.6605044

**Published:** 2009-04-14

**Authors:** L J Coleman, M B Peter, T J Teall, R A Brannan, A M Hanby, H Honarpisheh, A M Shaaban, L Smith, V Speirs, E T Verghese, J N McElwaine, T A Hughes

**Affiliations:** 1 Leeds Institute of Molecular Medicine, Leeds University, Leeds LS9 7TF, UK; 2 Department of Surgery, LGI, Leeds LS1 3EX, UK; 3 Department of Histopathology, SJUH, Leeds LS9 7TF, UK; 4 Department of Applied Mathematics and Theoretical Physics, Cambridge University, Cambridge CB2 0WA, UK

**Keywords:** translation factors, predictive biomarkers, Cox regression, pathway biomarker

## Abstract

Increased eukaryotic translation initiation factor 4E (eIF4E) expression occurs in many cancers, and makes fundamental contributions to carcinogenesis by stimulating the expression of cancer-related genes at post-transcriptional levels. This key role is highlighted by the facts that eIF4E levels can predict prognosis, and that eIF4E is an established therapeutic target. However, eIF4E activity is a complex function of expression levels and phosphorylation statuses of eIF4E and eIF4E-binding proteins (4E-BPs). Our hypothesis was that the combined analyses of these pathway components would allow insights into eIF4E activity and its influence on cancer. We have determined expression levels of eIF4E, 4E-BP1, 4E-BP2 and phosphorylated 4E-BP1 within 424 breast tumours, and have carried out analyses to combine these and relate the product to patient survival, in order to estimate eIF4E activity. We show that this analysis gives greater prognostic insights than that of eIF4E alone. We show that eIF4E and 4E-BP expression are positively associated, and that 4E-BP2 has a stronger influence on cancer behaviour than 4E-BP1. Finally, we examine eIF4E, estimated eIF4E activity, and phosphorylated 4E-BP1 as potential predictive biomarkers for eIF4E-targeted therapies, and show that each determines selection of different patient groups. We conclude that eIF4E's influence on cancer survival is modulated substantially by 4E-BPs, and that combined pathway analyses can estimate functional eIF4E.

The eukaryotic translation initiation factor 4E (eIF4E) has key roles in carcinogenesis (De Benedetti and Graff, 2004). eIF4E is often overexpressed in carcinoma cells as compared to equivalent normal epithelium in many tumour types including breast (Kerekatte *et al*, 1995), lung (Rosenwald *et al*, 2001) and colon (Rosenwald *et al*, 1999). The oncogenic role of this overexpression has been shown by various experimental observations; for example, forced eIF4E overexpression within many cell types leads to transformation (De Benedetti and Graff, 2004), and within transgenic mice increases incidence of multiple tumour types (Ruggero *et al*, 2004). eIF4E has at least two normal cellular functions. First, it is an essential component of the multimeric factor eIF4F, which initiates cap-dependent translation – the mechanism responsible for most protein synthesis (Gray and Wickens, 1998). eIF4E's role is to bind to mRNA caps allowing recruitment of eIF4F, and subsequently the translational machinery. The complex formed scans linearly along the 5′ untranslated region (UTR) until an initiation codon in good context is encountered, at which point further elements of translational machinery are recruited and protein synthesis starts. Second, eIF4E regulates expression of some genes by controlling nuclear export of their transcripts (Culjkovic *et al*, 2007), a function that also requires eIF4E's cap-binding activity (Culjkovic *et al*, 2005). Under most normal conditions, availability of active eIF4E is thought to be rate limiting for both functions. One might expect a general translational stimulation to result from the increased eIF4E expression in cancers, on account of enhanced mRNA cap recognition, yet effects of eIF4E overexpression are more subtle. Approximately 10% of mammalian transcripts have 5′UTRs that may form complex secondary structures that reduce the abilities of both eIF4F to bind to mRNAs and the translational machinery to scan 5′UTRs (Pesole *et al*, 2001); the result is that these transcripts are translated inefficiently (Hughes, 2007). The majority of human transcripts with these inhibitory 5′UTRs code for growth or cancer-associated proteins (Kozak, 1991). Increased eIF4E is thought to reduce the effects of 5′UTR structure by enhancing cap-recognition and scanning, therefore increasing translation of these specific oncogenic transcripts (De Benedetti and Graff, 2004). Similarly, increased eIF4E expression enhances nuclear export of a set of transcripts associated with oncogenesis (Culjkovic *et al*, 2007). As a consequence of this central role, eIF4E is an established target for cancer therapy (Smolewski, 2006; Graff *et al*, 2008).

The importance of eIF4E in cancer has been underlined by the fact that eIF4E expression levels can be used to determine prognosis. Cases in which eIF4E is highly overexpressed tend to have poor prognoses (Li *et al*, 1998). A substantial confounding factor is that eIF4E expression does not equate to eIF4E activity, thereby making interpretation of potential influences of eIF4E levels difficult. eIF4E activity is a complex function of eIF4E expression and expressions and activities of eIF4E-binding proteins (4E-BP1, 2 and 3) that bind to and inhibit eIF4E (Richter and Sonenberg, 2005) (Supplementary Figure S1). Activity is further regulated by phosphorylation of 4E-BP1 (and other 4E-BPs it is assumed), with only hypophosphorylated forms being able to inhibit eIF4E. Additional regulation occurs by differential phosphorylation of eIF4E itself, although there are conflicting reports as to how this influences activity (Scheper and Proud, 2002). The result is that high expression of eIF4E may not lead to high eIF4E activity if, for example, hypophosphorylated 4E-BP1 were also highly expressed. Many cancer-related signalling pathways, including PI3K and p38, converge to regulate eIF4E and 4E-BP phosphorylation; therefore, eIF4E activity seems to be a key cancer-signalling node (Polunovsky and Bitterman, 2006). Here, we have tested the hypothesis that combined analyses of expressions and phosphorylation states of eIF4E, and its regulators allows greater understanding of eIF4E activity and its influence on cancer than examination of eIF4E expression alone.

## Materials and methods

### Patients

Ethical approval was obtained (Leeds East 05/Q1206/136). Archival cancer tissue and data were obtained for 424 patients diagnosed at LTH NHS Trust from 1983–2006. Tissue microarrays (TMAs) were constructed containing 0.6 mm cores selected from representative tumour areas as determined by a consultant breast histopathologist (AMS) from H&E stained sections. Survival periods – overall: initial diagnosis to death; disease-free: initial diagnosis to the diagnosis of recurrence/metastasis; disease-specific: initial diagnosis to death after recurrence or metastasis (cancer-specific death confirmed in most cases).

### Westerns and immunohistochemistry

MCF7 and MDA-MB-231 cells were cultured/transfected as earlier (Johnson *et al*, 2008; Maraqa *et al*, 2008). An eIF4E expression vector was obtained from John Blenis (Harvard Medical School) and Nahum Sonenberg (McGill). Western analyses were carried out as earlier (Maraqa *et al*, 2008) using the reagents in Supplementary Table S1. TMA sections of 5 *μ*m were dewaxed and blocked in hydrogen peroxidase block (20 min). Antigens were retrieved and stained as described in Supplementary Table S1, and as used elsewhere (Zhou *et al*, 2004; Dutton *et al*, 2005; Lee *et al*, 2005; Engelman *et al*, 2008). Envision detection was used (DAKO, Glostrup, Denmark). Negative controls (primary antibodies omitted) were included in each immunohistochemistry (IHC) batch; in addition, adjacent normal epithelium, lymphocytes and blood vessel endothelium served as internal controls. Controls were performed for p4E-BP1 antibodies in which sections were pretreated with Lambda Phosphatase (Nebraska, NE, USA). Cores were scored for immunoreactivity by two or more individuals (LJC, TJT, ETV and RAB), taking into account the average intensity and percentage of positively stained tumour cells (as used earlier for eIF4E (Zhou *et al*, 2006)). Staining intensity scores (0 no staining, 1 weak, 2 moderate and 3 strong) were added to percentages positively stained scores (1 <5%; 2, 6–25%, 3, 26–75% and 4 >75%), giving totals of 0 or 2–7. Consensus scores were determined for cores with different initial scores, and all scoring was overseen by a consultant breast histopathologist (AMH).

### Mathematical analyses

Data were analysed using Kaplan–Meier survival curves. Dependence on prognostic indicators was determined using Cox proportional hazards models; significance values relate to likelihood ratio tests of the null hypothesis that indicators do not effect hazard rates (Cox and Oakes, 1984). SPSS (SPSS, Chicago, IL, USA) and the Statistics Toolbox in MATLAB (ecdf.m and coxphfit.m) (MathWorks, Natick, MA, USA) were used. Tests were two sided and *P*<0.05 was considered significant.

## Results

### Antibody validation

We have used IHC to determine expression levels in breast tumours of the main regulatory molecules of the eIF4E pathway – namely, eIF4E, 4E-BP1, 4E-BP2 and phosphorylated 4E-BP1 (Thr37/46) (termed p4E-BP1). We have not examined 4E-BP3 because it is not thought to have a role in breast (Poulin *et al*, 1998), or phosphorylated forms of eIF4E, as their influences on the activity and in cancer remain uncertain (Scheper and Proud, 2002; Salehi and Mashayekhi, 2006; Buxade *et al*, 2008). First, we optimised the antibody use on archival breast tissue. We established that antibodies were specific for their antigens using western blots against lysates of breast cancer cell lines (Supplementary Figure S2A). In addition, we showed phospho-specificity of antibodies against p4E-BP1 by carrying out IHC on serial tissue sections with and without pretreatment with protein phosphatase (Supplementary Figure S2B).

### Patient cohort and immunohistochemistry

**Figure 1:**
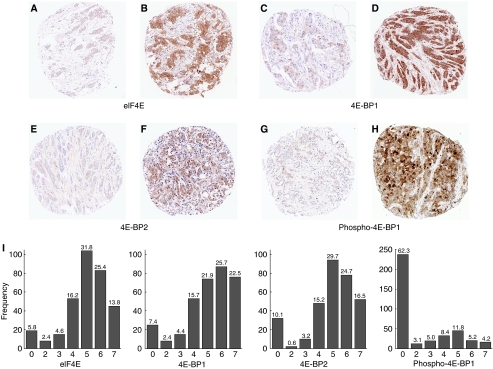
The full range of expression intensities and proportions for eukaryotic translation initiation factor 4E (eIF4E), eIF4E-binding protein (4E-BP) 1, 4E-BP2 and p4E-BP1 occur within breast cancers. (**A**–**H**) Representative tumour tissue microarray cores showing immunoreactivity as labelled. These cores were scored A 4, B 7, C 3, D 7, E 3, F 7, G 3 and H 7. (**I**) Histograms showing distributions of immunohistochemistry scores within breast cancers. Scores (x-axis) and numbers of cores assigned to each score (y-axis) are shown. Percentages of the cohort are given above the bar for each score.

Tissue micro-arrays containing samples from 424 breast tumours were established, supported by detailed clinicopathological data (Supplementary Table S2). The cohort included a wide range of patient and tumour characteristics, with mean patient follow-up of 91.9 months. We carried out IHC for eIF4E, 4E-BP1, 4E-BP2 and p4E-BP1 on TMA sections and assessed immunoreactivity within tumour cells, taking into account the proportions of cells staining positively and average intensity, giving scores of 0 (negative) or 2–7 (positive). Representative staining patterns are shown (Figure 1, and at higher magnification in Supplementary Figure S3). Tumour stroma and normal tissue were negative for eIF4E, 4E-BP1 and 4E-BP2, whereas very occasional low-intensity staining for p4E-BP1 was noted in normal epithelial cells. Staining was generally cytoplasmic, although nuclear staining was noted in a minority of cases (Supplementary Figure S4); this was separately analysed and was found not to be of prognostic value and is not discussed. As expected, data were not available for some patients because of the TMA core loss during processing, a well-recognised occurrence, therefore, data for all four antigens were available for only 282 patients. The full range of scores were observed for each antigen (Figure 1I). It was notable that staining was most frequently not detectable for p4E-BP1. Others have reported more frequent expression of p4E-BP1 (Zhou *et al*, 2004), therefore we carried out IHC for an alternative p4E-BP1 species (Ser65); we found immunoreactivity with this antibody to be similarly infrequent (see discussion).

### Expressions of eIF4E and 4E-BPs correlate with grade

Associations between antigen expressions and a wide range of clinicopathological parameters were examined. No correlations were found with nodal status, tumour size or histological type. Weak positive/borderline no correlations were found with oestrogen receptor *α* status and eIF4E expression (Spearman's *ρ* coefficient 0.21; *P*<0.001) and 4E-BP2 (0.22; *P*<0.001), but not with 4E-BP1 or p4E-BP1. Strong correlations between expression of markers and tumour grade were found. eIF4E expression (split into three classes, 0–3, 4–5 and 6–7) was positively associated with grade (*χ*^2^-test, *P*=0.011), whereas expression of both 4E-BPs was negatively associated with grade (4E-BP1 *P*=0.002; 4E-BP2 *P*=0.029). p4E-BP1 was positively associated with grade (*P*=0.012). A positive association between eIF4E expression and grade has been reported earlier (Li *et al*, 2002). Correlations for other markers were consistent with their influences on carcinogenesis being through the eIF4E pathway; 4E-BPs, eIF4E inhibitors, were negatively associated with grade, whereas 4E-BP1 phosphorylation, which would relieve 4E-BP1-induced inhibition of eIF4E, was positively associated.

### High expression of eIF4E correlates with poor prognosis

**Figure 2:**
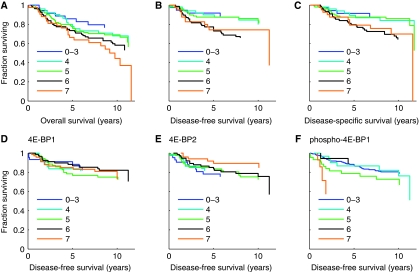
Expression of eukaryotic translation initiation factor 4E (eIF4E), but not eIF4E-binding protein (4E-BP) 1, 4E-BP2 or p4E-BP1, is associated with prognosis. (**A**–**C**) Kaplan–Meier survival analyses for overall (**A**), disease-free (**B**) and disease-specific survival (**C**) for patient groups with tumours with differing eIF4E scores. (**D**–**F**) Kaplan–Meier survival analyses for disease-free survival for patient groups with tumours with differing scores for 4E-BP1 (**D**), 4E-BP2 (**E**) or p4E-BP1 (**F**). As scores of 0, 2 and 3 were relatively rare, these have been grouped together. Censoring ticks have been omitted for clarity.

Kaplan–Meier survival analyses were used to determine survival with respect to eIF4E. Analyses were carried out with expression divided into IHC scores, although scores of 0, 2 and 3 were combined as each individual group was small, for overall survival (OS), disease-free survival (DFS) and disease-specific survival (DSS) (Figure 2A–C). High eIF4E scores were indicative of poor prognosis. Prognosis seemed to worsen with each increasing eIF4E score for OS, whereas patterns for DFS and DSS suggested weaker, but still detectable, influences of individual scores with an overall grouping into two classes (0–5 good prognosis; 6 or 7 poor prognosis). We have examined relationships between eIF4E expression and survival using Cox regressions. We have included either eIF4E scores, or eIF4E expression dichotomised arbitrarily or as suggested by the apparent bimodal distribution seen above, and modelled these with respect to OS, DFS and DSS. Models that most accurately reflected the data included eIF4E scores rather than dichotomised data, showing the value of scoring proportion and intensity of positive tumour cells. In these models each increase of 1 in eIF4E score gave increases in hazard ratios (HRs) of 1.22 (*P*=0.004), 1.3 (*P*=0.008) and 1.33 (*P*=0.005) for OS, DFS and DSS, respectively. Thus individuals with scores of 7 have DFS HRs of 6.15 (95% CIs: 3–12, *P*=0.008) as compared with individuals with scores of 0. The prognostic value of eIF4E has been reported earlier as independent of grade/nodal status in breast cancer (Li *et al*, 1998); we have examined independence from the Nottingham Prognostic Index (NPI), which takes account of tumour size, grade and lymph node status (Haybittle *et al*, 1982). In multivariate Cox regressions the prognostic value of eIF4E was independent of NPI with eIF4E remaining significant for OS (NPI *P*<0.00001; eIF4E *P*=0.02), DFS (NPI *P*<0.00001; eIF4E *P*=0.045) and DSS (NPI *P*<0.00001; eIF4E *P*=0.029).

### Prognostic value of 4E-BP1, 4E-BP2 and p4E-BP1

Kaplan–Meier survival analyses were also used to determine survival with respect to the other markers. We present data for DFS (Figure 2D–F), and for OS and DSS (Supplementary Figure S5). These antigens provided little prognostic insight and we were unable to construct significant Cox equations to model their individual survival influences. In the case of 4E-BP2, there was a nonsignificant trend for high scores to associate with good prognosis.

### Mathematical modelling of influences of 4E-BPs: 4E-BPs modify eIF4E activity and provide additional prognostic insights

Next we examined influences of 4E-BPs in the context of eIF4E expression. We necessarily restricted these analyses to the 282 patients for whom scores of all four antigens were available. Using this dataset, models including solely eIF4E expression gave HRs of 1.21 (*P*=0.011), 1.24 (*P*=0.035) 1.27 (*P*=0.02) for OS, DFS and DSS, respectively, for each increase of 1 in eIF4E score. We were unable to construct significant Cox models on the basis of combinations of 4E-BPs without including eIF4E, suggesting that eIF4E is their critical effector. We found models combining expression of 4E-BP1 or p4E-BP1 (as IHC scores, or dichotomised into two groups) with eIF4E, provided little additional prognostic value over that found with eIF4E alone. However, including 4E-BP2 in a model for OS enhanced the model significantly with hazard increasing by 1.28 with each point increase in eIF4E score (*P*=0.005) and decreasing 0.11 with each increase in 4E-BP2 score (*P*=0.02). We refined this by combining eIF4E and 4E-BP2 scores into a single non-linear variable in which high levels of eIF4E or 4E-BP2 act to increase or decrease the value respectively (achieved using max (0,X-B2/3.5), where X and B2 represent eIF4E and 4E-BP2 scores). This variable, termed ‘*y*’, predicted survival more accurately than examination of eIF4E alone; each increase of 1 in *y* carried HRs of 1.32 (*P*=0.0003), 1.32 (*P*=0.013) and 1.36 (*P*=0.006) for OS, DFS and DSS, respectively. We also investigated Cox models including expression of all four antigens. In order to combine terms, we considered models that included each variable separately and found their relative effect on HRs using maximum likelihood estimation. Although individual components were not statistically significant and had only little effects on likelihoods (with the exception of eIF4E), a combination gave improved prognostic power. This variable termed ‘*z*’ can be described as X–B1/4+PB1/2-B2/4, where B1 and PB1 represent 4E-BP1 and p4E-BP1. Each increase of 1 in *z* gave HRs of 1.15 (*P*=0.006), 1.26 (*P*=0.002) and 1.28 (*P*=0.0008) for OS, DFS and DSS, respectively. This variable has a highly significant relationship with survival but this should be treated with caution because the constants were determined using regressions for OS, and, thus to an extent, significance is self fulfilling, at least for OS. The utility of *z*, however, is supported by the fact that its relationship with survival is more significant with DFS and DSS than OS, a result not predetermined by the approach. We also examined whether *y* or *z* give prognostic insights independently of NPI using multivariate analyses. NPI and either *y* or *z* remain significant in models for DFS (NPI *P*<0.00001; *y P*=0.04 or *z P*=0.03) and DSS (NPI *P*<0.00001; *y P*=0.02 or *z P*=0.02).

**Figure 3:**
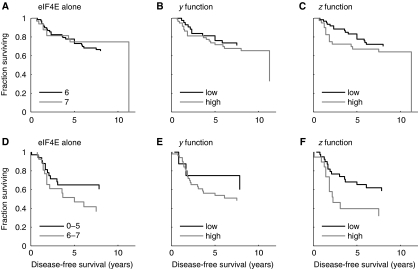
Additional prognostic information is gained by combining assessment of eukaryotic translation initiation factor 4E (eIF4E)-binding proteins (4E-BPs) with eIF4E analysis. Kaplan–Meier survival analyses of disease-free survival for patients with either high (6 or 7) eIF4E scores (**A**–**C**) or high (>5.4) Nottingham Prognostic Index scores (**D**–**F**). Patients were dichotomised using either eIF4E expression (**A** and **D**), the *y* function (which includes eIF4E and 4E-BP2 scores) (**B** and **E**), or the *z* function (which includes scores for all 4 markers) (**C** and **F**).

The statistical significance of relationships of *y* and *z* with survival show additional prognostic value from examining multiple eIF4E pathway components. In addition, we have shown the value of these variables using Kaplan–Meier analyses. First, we focused on patients with high eIF4E scores (6 or 7), as it is in this context that differential expression of 4E-BPs would be most relevant. Patients with eIF4E scores 6 or 7 have a relatively poor prognosis (Figure 2A–C), but no difference was detected between groups scored as 6 or 7 in terms of DFS (Figures 2B and 3A). When *y* was applied to this cohort some discrimination occurred with improved prognosis for patients whose *y* scores were lowered by 4E-BP2 (Figure 3B), although the discrimination remained statistically nonsignificant. When *z* was applied to this cohort further discrimination occurred (Figure 3C) showing how 4E-BPs affect patient outcome through eIF4E. Second, we have focused on patients with high NPI (and consequently poor DFS, Supplementary Figure S6). These patients were further stratified according to eIF4E expression (cutoff 5.5 as suggested by the distribution in Figure 2B) into separate groups (Figure 3D). As before, when *z* was applied (Figure 3F) further discrimination occurred allowing identification of patients with very poor (high *z*), or relatively good prognosis (low *z*). In this case, *z* discriminated into statistically significantly different groups (Figure 3F Log rank *P*=0.039) when use of eIF4E alone was not significant (Figure 3D Log rank *P*=0.15). In this case *y* was substantially less successful as a prognostic indicator (Figure 3E Log rank *P*=0.5) showing the importance of combining all four components.

### Expressions of eIF4E and 4E-BPs are positively associated

Although including assessments of 4E-BPs provided additional prognostic information over that from only eIF4E, we were surprised that influences of 4E-BPs, especially of 4E-BP1, were relatively weak. One explanation for this was that expression levels of eIF4E and 4E-BPs were not independent of each other. This would mean that that at a given eIF4E level, differential expression of 4E-BPs – therefore differential modification of eIF4E activity – would be relatively rare, thereby minimising apparent influences of 4E-BPs in our analyses. Associations between marker expressions were examined using Spearman's correlation tests (Table 1). eIF4E expression showed moderate positive associations with expression of both 4E-BPs (*P*<0.0001), although weak positive/no association with p4E-BP1. Expression of 4E-BPs was also moderately positively associated with each other (*P*=0.02). Expression of p4E-BP1 was positively associated with 4E-BP1 (*P*<0.0001) (expected as 4E-BP1 must be expressed to be phosphorylated).

### eIF4E activity scores are potential biomarkers for eIF4E-targeted therapies

**Figure 4:**
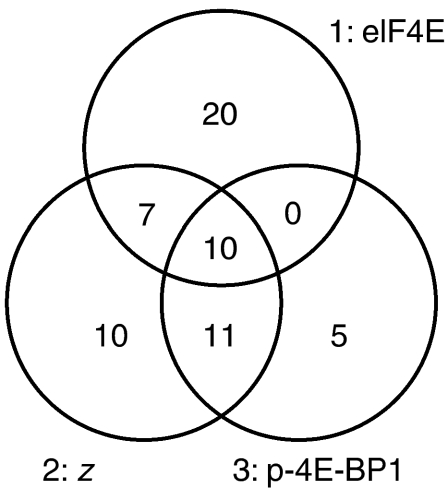
Use of biomarkers for dividing patients into potential treatment groups for eukaryotic translation initiation factor 4E (eIF4E)-directed therapy; different markers select very different groups. A Venn diagram demonstrating relationships between potential treatment groups selected on the basis of high eIF4E expression (1), high estimated eIF4E activity (‘*z*’) (2), and high p4E-BP1 expression (3).

The eIF4E pathway is a target for cancer therapy with drugs that either inhibit eIF4E activity indirectly (mTOR inhibitors that reduce 4E-BP1 phosphorylation thereby inhibiting eIF4E (Smolewski, 2006)), or directly (by binding to/reducing expression of eIF4E (Graff *et al*, 2008)). However, a concern with these agents is toxicity resulting from general translational repression. Consequently, selection of patients who are most likely to benefit from such agents using predictive biomarkers may aid their efficacy. Three selection criteria are apparent: individuals with highest eIF4E levels, highest eIF4E activities or highest levels of phosphorylated 4E-BP1 (especially relevant for mTOR inhibitors as these act by reducing 4E-BP phosphorylation). We have compared selection of potential treatment groups using these criteria from our cohort for whom scores of all four antigens were available: first (group 1), those with eIF4E scores of 7 (37/282; 13.1%); second (group 2), those with high estimated pathway activities (z⩾5.75, cutoff chosen to give a similar-sized group, 38/282; 13.5%); finally (group 3), those with p4E-BP1 scores of 6 or 7 (26/282; 9.2%). We have examined whether biomarker choice leads to selection of different potential treatment groups (Figure 4). Substantially different patient groups were selected using each potential predictive biomarker. Only 10 patients appeared in all groups (16% of total patients selected in any group). Group 1 contained the largest proportion of uniquely selected individuals (56% of total), whereas the majorities of groups 2 and 3 (74 and 81% respectively) overlapped with at least one other group. Group 2 (high *z* values) had large overlaps with both other groups (46% of group 1 and 81% of group 3). In addition, group 2 included all individuals with high eIF4E *and* p4E-BP1 (i.e. individuals likely to have high eIF4E activity by all measures) reflecting the fact that *z* successfully takes account of both eIF4E and p4E-BP1, thereby supporting its utility as a potential predictive marker.

## Discussion

Expression of eIF4E in cancer has been studied extensively, however, expression does not equate to activity; therefore, interpretation of its influence is more complex than simply assessing expression. Our hypothesis was that combined examination of eIF4E and its regulators would allow greater insights into eIF4E's influence on cancer. Therefore, we determined the expression levels of eIF4E and its most well-established regulatory proteins 4E-BP1, 4E-BP2 and p4E-BP1 within tumour cells of a large cohort of cancer patients, and have combined these data into an improved measure of prognosis and estimate of eIF4E activity. In common with initial publications on eIF4E's role in cancer (Kerekatte *et al*, 1995; Li *et al*, 1998) and much of the subsequent literature, we have focused on breast cancer.

There is a wealth of literature on eIF4E expression in cancer and, although patient cohorts used have been relatively small (<200 individuals), the conclusion that high eIF4E levels are associated with poor prognosis is well established (De Benedetti and Graff, 2004). We have also found this (Figure 2A–C) using the largest cohort to date. The 4E-BPs have received less attention. 4E-BP1 is more highly expressed in tumours than normal tissues (Salehi and Mashayekhi, 2006), and expression correlates inversely with tumour progression (Martin *et al*, 2000) – observations in agreement with our findings. Despite the inverse correlation with progression, we found examination of 4E-BP1 expression not to give significant prognostic insights (Figure 2D, Supplementary Figure S5A). To our knowledge, there are no published studies concerning 4E-BP2 in cancer samples. We found that 4E-BP2 showed a nonsignificant trend for association with good prognosis (Figure 2E, Supplementary Figure S5B). High p4E-BP1 levels have earlier been shown to correlate with grade and poor prognosis (Zhou *et al*, 2004; Castellvi *et al*, 2006; Rojo *et al*, 2007). In contrast, we found detectable p4E-BP1 (Thr37/46) in only 37.7% of the patients compared with >59% in other reports (Zhou *et al*, 2004; Rojo *et al*, 2007; Akcakanat *et al*, 2008)), and to provide little prognostic power when analysed alone (Figure 2F, Supplementary Figure S5C). We have carried out additional analyses with the same antibody as reported earlier for p4E-BP1 (Ser65) (Zhou *et al*, 2004) and similarly found low expression (50% undetectable, 28% the lowest positive score). These differences may relate to the cohorts used as earlier studies had higher proportions of node positive (Zhou *et al*, 2004), subsequently metastatic (Akcakanat *et al*, 2008) or high-grade cases (Rojo *et al*, 2007).

There are very few studies where multiple eIF4E pathway components have been analysed. The ratios of p4E-BP1 to total 4E-BP1 and of eIF4E to 4E-BP1 have been shown to correlate with high tumour grade (Salehi and Mashayekhi, 2006; Armengol *et al*, 2007), but their relationships with survival were not examined. A positive correlation between expression of eIF4E and p4E-BP1 has also been noted, with the conclusion being that eIF4E was ‘active’ in these cells (Nathan *et al*, 2004). We have determined expression levels of eIF4E, 4E-BP1, 4E-BP2, p4E-BP1 and have undertaken analyses to relate these together and to survival. First, we found no statistically significant relationship to survival for any combination without including eIF4E – suggesting that eIF4E is the critical effector. Second, in support of our initial hypothesis, we showed that combined assessment of the four components allowed improved prognostic insights over eIF4E alone (‘*z*’ function; text, Figure 3). It is important to note that we did not predetermine relationships between components in *z*, rather these were defined by the best fit with the data; the fact that the relationships reflect our expectations from understanding the pathway supports the view that *z* is a true estimate of eIF4E activity. Third, we showed differential expression of 4E-BP2 in cancer to be more influential in terms of survival than 4E-BP1. This was shown by the observations that expression of 4E-BP2, but not 4E-BP1, showed a trend towards being a prognostic factor alone (Figure 2E, Supplementary Figure S5B), provided an improved prognostic indicator in combination with eIF4E (*y*), and was the most statistically significant component of *z* after eIF4E itself. This observation may relate to the fact that 4E-BP2 binds, and therefore inhibits eIF4E more strongly than 4E-BP1 (Abiko *et al*, 2007). Interestingly, we found that expressions of eIF4E and 4E-BPs were positively associated (Table 1): an unexpected finding as they are functionally opposed and correlate oppositely with grade. One explanation is that 4E-BP translation may be specifically derepressed by eIF4E's action on the 5′UTRs of their transcripts, representing a negative feedback loop within the eIF4E pathway.

Clinical trials of the efficacy and safety of cancer therapeutics that target eIF4E have been carried out (Graff *et al*, 2008) and some toxicity has been reported (O'Donnell *et al*, 2008; Tabernero *et al*, 2008). Selection of individuals who are most likely to benefit from the agents may be appropriate in order to avoid potentially harmful and/or ineffective therapy in some patients. We show that substantially different patient groups are chosen using three potential predictive biomarkers, and therefore that use of the best biomarker is important for targeting of these therapies (Figure 4). Patients with high estimated eIF4E activity (‘*z*’) (group 2) and high p4E-BP1 levels (group 3) should provide good candidates for treatment. The former group has a particularly poor prognosis (Figure 3C and F), and therefore great potential for clinical benefit from these drugs.

## Figures and Tables

**Table 1:** Expressions of eIF4E, 4E-BP1 and 4E-BP2 are positively associated

	**eIF4E**	**4E-BP1**	**4E-BP2**	**p4E-BP1**
eIF4E	—	0.31	0.34	0.21
4E-BP1	0.31	—	0.37^*^	0.36
4E-BP2	0.34	0.37^*^	—	0.14
P4E-BP1	0.21	0.36	0.14	—

Abbreviations: eIF4E=eukaryotic translation initiation factor 4E; 4E-BP=eIF4E-binding protein; P4E-BP1=phosphorylated 4E-BP1.

Spearman's *ρ* correlation figures are shown. Associations are either moderately (^*^P=0.02) or highly significant (others *P*<0.0001).
