# Supplementary figures and images for: Combined analysis of eIF4E and 4E-binding protein expression predicts breast cancer survival and estimates eIF4E activity
Source: Br J Cancer. 2009 Apr 14;100(9):1393–9. doi: 10.1038/sj.bjc.6605044 (PMC2694424; doi:10.1038/sj.bjc.6605044)

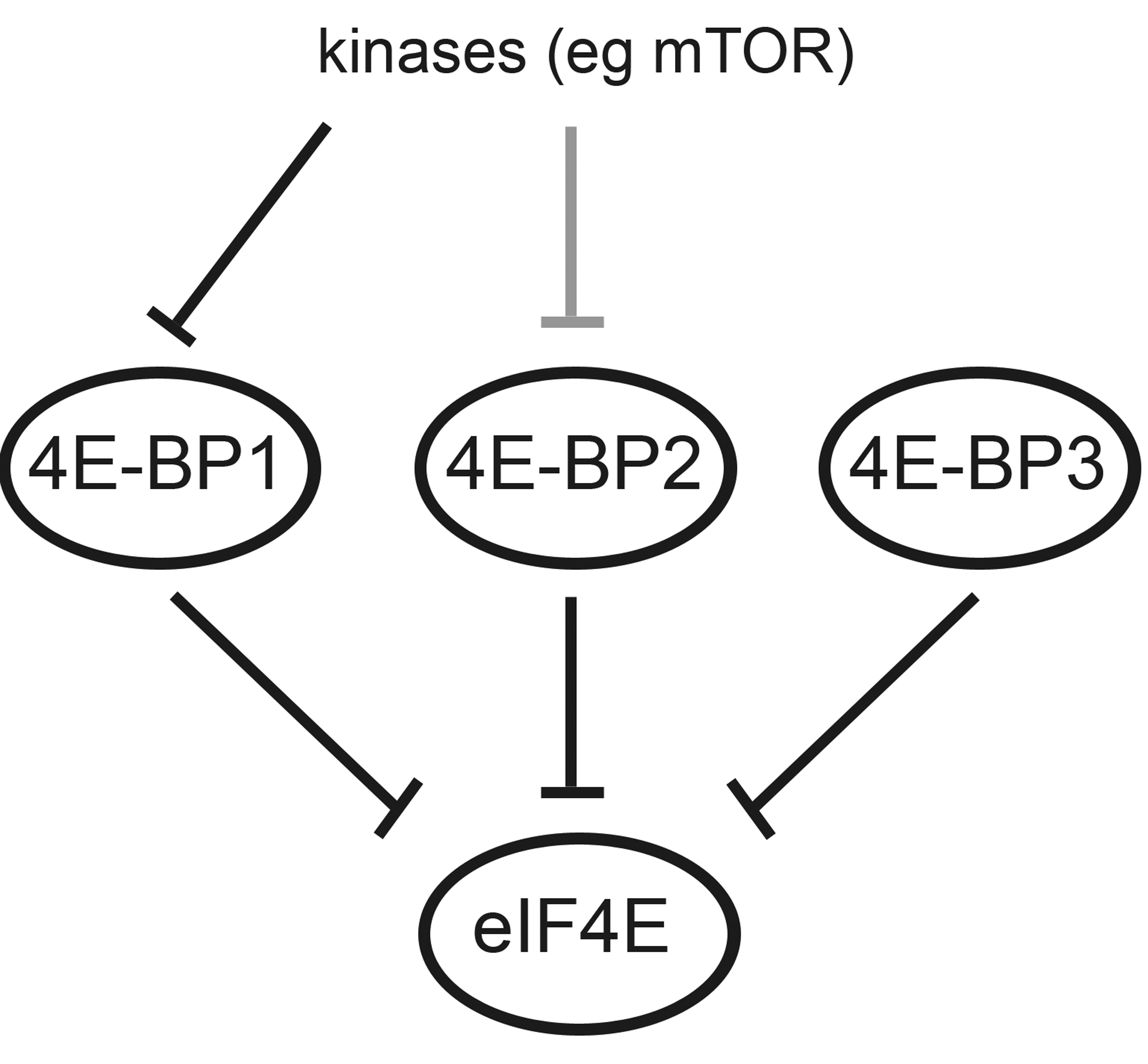

Supplement: Supplementary Figure S1 [file 6605044x1.tif]

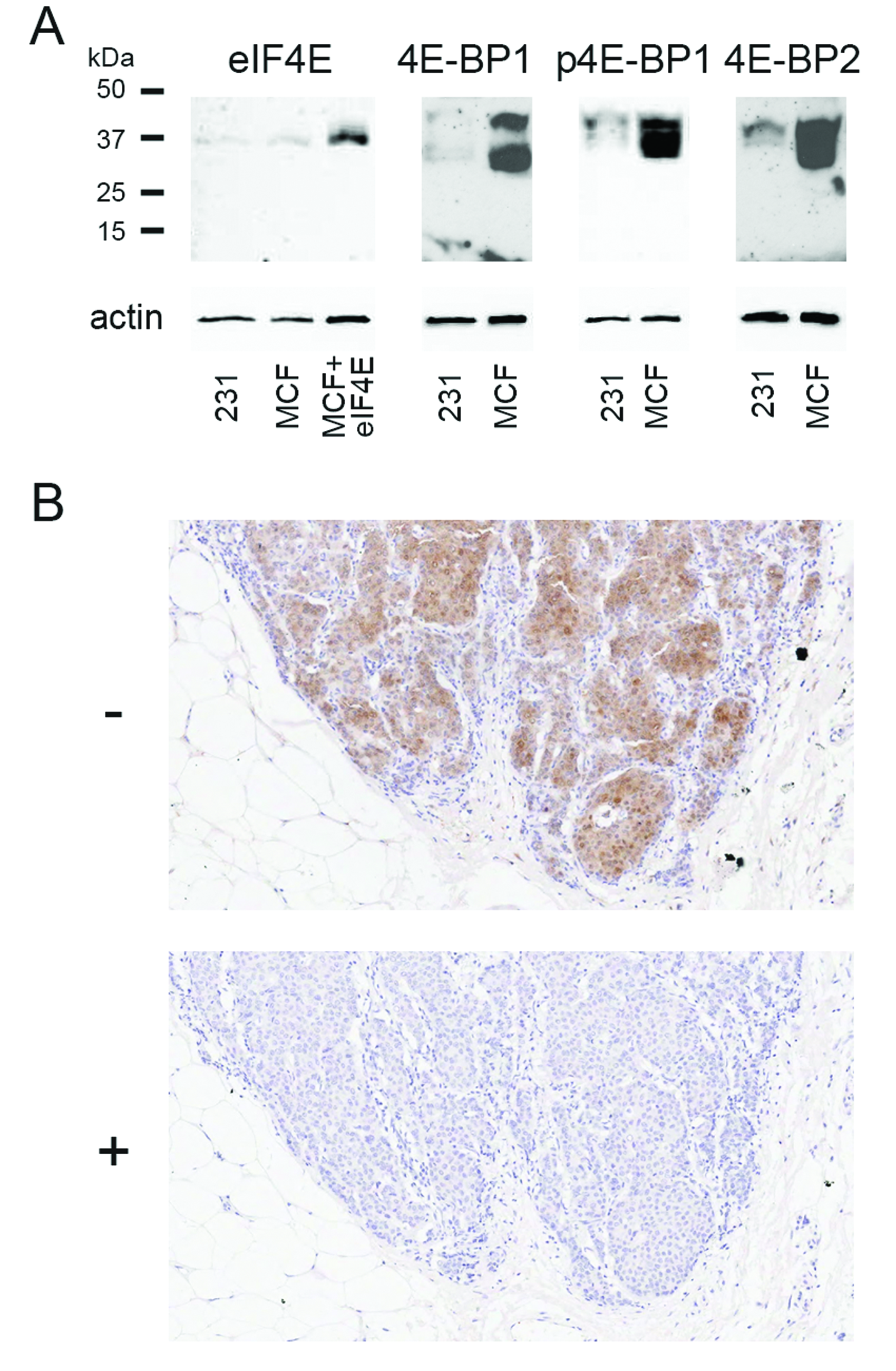

Supplement: Supplementary Figure S2 [file 6605044x2.tif]

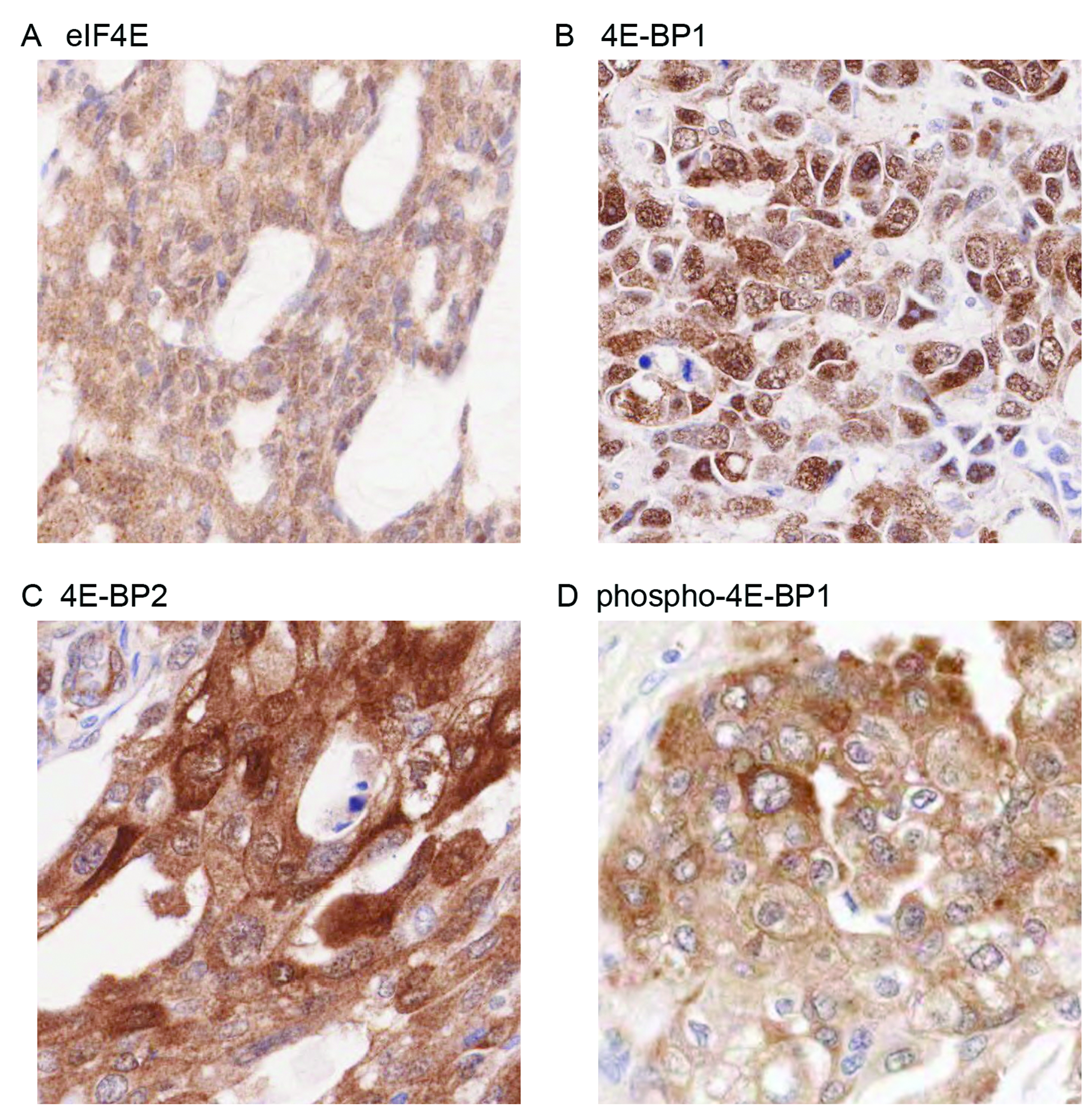

Supplement: Supplementary Figure S3 [file 6605044x3.tif]

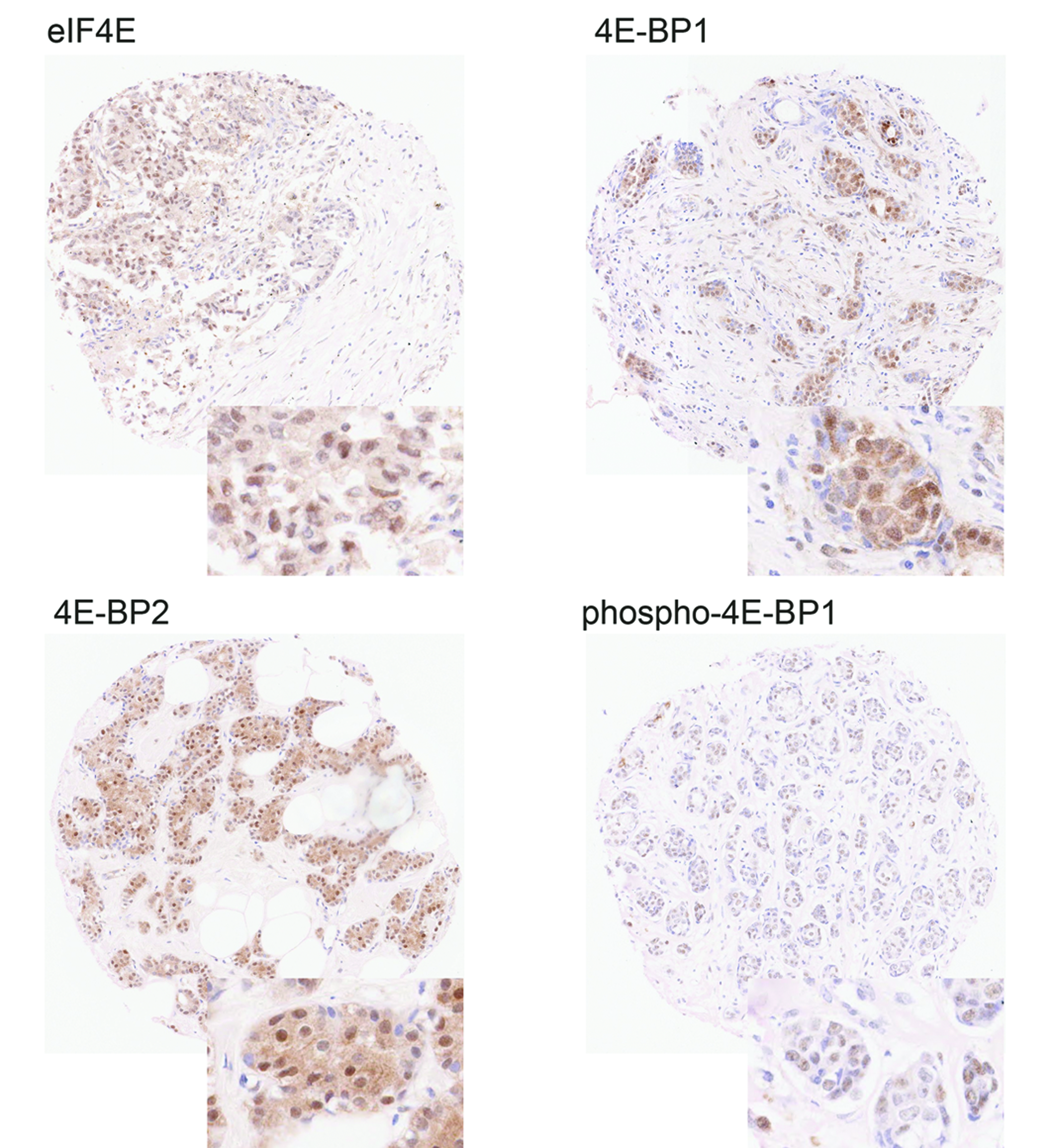

Supplement: Supplementary Figure S4 [file 6605044x4.tif]
