# Supplementary material for: Combined analysis of eIF4E and 4E-binding protein expression predicts breast cancer survival and estimates eIF4E activity
Source: Br J Cancer. 2009 Apr 14;100(9):1393–9. doi: 10.1038/sj.bjc.6605044 (PMC2694424; doi:10.1038/sj.bjc.6605044)
Supplement: Supplementary Table S1 [file 6605044x7.doc]

| **Antigen** | **Antibody** | **Westerns**:  antibody dilution (4°C overnight) | **IHC**:  antigen retrieval; antibody dilution (all 4°C overnight) |
| --- | --- | --- | --- |
| eIF4E | mouse monoclonal sc9976, Santa Cruz, USA | 1:500 | 2 min pressure cooker (boiling /maximum pressure) in antigen un-masking solution (Vector, USA); 1:100 |
| 4E-BP1 | rabbit polyclonal #9452, CST, USA | 1:1000 | no antigen retrieval; 1:100 |
| 4E-BP2 | rabbit polyclonal #2845, CST, USA | 1:2000 | 12 min full-power microwave in pH6 citrate buffer; 1:100 |
| p4E-BP1 Thr37/46 | rabbit monoclonal #2855, CST, USA | 1:1000 | 12 min full-power microwave in pH6 citrate buffer; 1:150 |
| p4E-BP1 Ser65 | rabbit polyclonal #9451, CST, USA | 1:1000 | 12 min full-power microwave in pH6 citrate buffer; 1:25 |

**Table S1** Reagents and conditions for Western and immunohistochemistry analyses.
