# Supplementary material for: Combined analysis of eIF4E and 4E-binding protein expression predicts breast cancer survival and estimates eIF4E activity
Source: Br J Cancer. 2009 Apr 14;100(9):1393–9. doi: 10.1038/sj.bjc.6605044 (PMC2694424; doi:10.1038/sj.bjc.6605044)
Supplement: Supplementary Table S2 [file 6605044x8.doc]

| **Characteristic** | **Details** | **Cases** |
| --- | --- | --- |
| Patients within cohort (number for whom full follow up data available) |  | 424  (408) |
| Age (years) | 27–92 (median 57) |  |
| Tumour size (mm) | 18–35 (median 27.5) |  |
| Lympho-vascular invasion | None  Present  Probable  Missing data | 205  194  4  5 |
| Positive axillary nodes |  | 186 |
| Tumour grade | 1  2  3 | 91  179  138 |
| Nottingham Prognostic Indicator (NPI) | 2.08–8 (median 4.3) |  |
| Histological tumour type | Ductal  Lobular  Mucinous  Tubular  Mixed | 321  44  10  5  28 |
| Recurrences |  | 13 |
| Metastases |  | 72 |
| Follow-up period (months) | 1–214 (mean 91.9, SD 42.21) |  |

**Table S2** Clinicopathological features of breast cancer cases included in this study.
