# Supplementary material for: Combined analysis of eIF4E and 4E-binding protein expression predicts breast cancer survival and estimates eIF4E activity
Source: Br J Cancer. 2009 Apr 14;100(9):1393–9. doi: 10.1038/sj.bjc.6605044 (PMC2694424; doi:10.1038/sj.bjc.6605044)
Supplement: Supplementary Figure legends [file 6605044x9.doc]

**Figure S1** eIF4E expression does not equate to eIF4E activity. eIF4E function is regulated by changes in expression of eIF4E and of 4E-BP1, 2 and 3, which bind to eIF4E and inhibit its activity. Further regulation occurs by phosphorylation of 4E-BP1 (and likely 2 and 3), with only hypophosphorylated forms being able to interact with eIF4E.

**Figure S2** Antibodies directed against eIF4E, 4E-BP1, 4E-BP2 and p4E-BP1 are specific for their targets. A) Western blots were performed on lysates of MDA-MB-231 (231) or MCF7 breast cancer cells (untransfected, MCF, or transfected with an expression vector for eIF4E, MCF+eIF4E). A protein corresponding to over-expressed eIF4E is detected with the anti-eIF4E antibody. Multiple protein species are detected with each 4E-BP antibody reflecting the varied mobilities of the different phosphorylated species*.* B) IHC for p4E-BP1 was performed on serial sections of breast cancer tissue with and without pre-treatment with protein phosphatase. Strong p4E-BP1 staining within tumour cells was seen in untreated sections while there was no appreciable staining within the same tissue area in treated sections.

**Figure S3** Representative high magnification images of breast tumour TMA cores showing strong immunoreactivity for eIF4E, 4E-BP1, 4E-BP2 and p4E-BP1.

**Figure S4** Representative breast tumour TMA cores (with magnified region) showing nuclear immunoreactivity for eIF4E, 4E-BP1, 4E-BP2 and p4E-BP1.

**Figure S5** Expression of 4E-BP1, 4E-BP2 and p4E-BP1 is not associated with prognosis. Kaplan-Meier survival analyses for overall survival (left panel) and disease-specific survival (right panel) for five different patient groups with tumours with differing levels of 4E-BP1 (A), 4E-BP2 (B) or p4E-BP1 expression (C).

**Figure S6** The Nottingham Prognostic Index (NPI) divides patient into groups with good, intermediate, or poor prognoses. Kaplan-Meier survival analyses for disease-free survival for patient groups with high (>5.4), intermediate (>3.4 and <=5.4) or low (<=3.4) NPI scores.
